# Oncogenic Role and Drug Resistance Effect of CCDC6 in iCCA: Potential Strategies for Targeted Intervention

**DOI:** 10.1111/jcmm.70855

**Published:** 2025-10-02

**Authors:** Jiaming Chen, Qiaoting Wu, Bodeng Wu, Guanbo Wang, Jiawei Li, Zhenxun Wang, Xinyi Tan, Bo Ma, Xiaoqing Jiang, Xin Zhang, Yu Wang

**Affiliations:** ^1^ Department of Hepatobiliary Surgery, Nanfang Hospital Southern Medical University Guangzhou P.R. China; ^2^ Department of Laboratory Medicine, Guangdong Provincial Key Laboratory of Precision Medical Diagnostics, Guangdong Engineering and Technology Research Center for Rapid Diagnostic Biosensors, Guangdong Provincial Key Laboratory of Single Cell Technology and Application, Nanfang Hospital Southern Medical University Guangzhou P.R. China; ^3^ Department of Clinical Medicine Guangdong Medical University Clinical College of Medicine Dongguan P.R. China; ^4^ Intensive Care Unit, Nanfang Hospital Southern Medical University Guangzhou P.R. China

**Keywords:** chemoresistance, coiled‐coil domain‐containing 6, DNA damage repair, epithelial‐mesenchymal transition, intrahepatic cholangiocarcinoma

## Abstract

Intrahepatic cholangiocellular carcinoma (iCCA) is a digestive neoplasm with a very poor prognosis. This study examined the role of CCDC6 in the progression of iCCA and gemcitabine resistance through bioinformatics analysis and functional assays. Database analysis showed that CCDC6 was significantly overexpressed in iCCA, with its expression positively correlating with TNM stage and lymph node metastasis. Although its value as an independent prognostic biomarker was not statistically significant in survival analysis, its expression profile suggested potential as a therapeutic target. Functional experiments demonstrated that CCDC6 knockdown inhibited iCCA cell proliferation, migration and invasion in vitro in a dose‐dependent manner, while overexpression enhanced these malignant phenotypes. Western blot analysis demonstrated that CCDC6 regulated epithelial‐mesenchymal transition (EMT) by modulating Vimentin and Snail expression, promoting metastasis. In vivo xenograft and metastasis models confirmed that CCDC6 depletion significantly reduced tumour burden and lung metastases. Moreover, CCDC6 and the EMT marker Vimentin were upregulated in gemcitabine‐resistant iCCA cells. γH2AX foci staining indicated that CCDC6 enhanced DNA damage repair (DDR), reducing chemotherapy‐induced genomic instability, while CCDC6 inhibition exacerbated DNA damage, reversing resistance. These findings suggest CCDC6 drives chemoresistance through EMT activation and enhanced DDR, making it a promising therapeutic target in iCCA.

## 1 Introduction

Intrahepatic cholangiocarcinoma (iCCA) is a malignant neoplasm that arises from the epithelial cells of small intrahepatic bile ducts or terminal bile ducts at the distal end of secondary bile ducts, representing approximately 20% of all primary liver malignancies [1]. During the past decade, epidemiological studies have demonstrated a significant upward trend in both incidence and mortality rates of iCCA globally [2, 3]. In contrast, the aetiology and pathogenesis of iCCA are poorly defined, and apart from resection, IDH and FGFR2 targeted biologic therapies, only platinum‐based and tamoxifen‐based drug therapies can slightly delay patient survival [4, 5]. The five‐year survival rate for iCCA patients continues to remain dismal at less than 20%, primarily due to high rates of tumour recurrence and chemoresistance [1, 6]. These clinical challenges underscore the urgent need for identifying novel causative genes and potential therapeutic targets.

Chemoresistance represents a significant obstacle in cancer treatment, with around 90% of chemotherapy patients ultimately experiencing relapse or metastasis due to acquired drug resistance [7]. Its causes are complicated, and aberrant enhancement of DNA damage repair (DDR) capacity in tumour cells leading to apoptotic escape is one of the common causes, which has been reported in lung cancer, cervical and BTC [8, 9, 10]. Besides, EMT significantly contributes to tumour progression by enhancing the invasiveness and anti‐apoptotic properties of cancer cells, which promote their resistance to treatment [11, 12, 13, 14].

CCDC6 is a member of the coiled‐coil domain‐containing (CCDC) protein family located on human chromosome 10q21, which has garnered increasing attention in cancer research [15]. It was initially noticed by researchers as a fusion gene aberrantly expressed in malignant tumours such as gastric and thyroid cancers [16, 17, 18]. In our previous work, a case of iCCA with metastatic lung nodules harbouring an FGFR‐CCDC6 fusion mutation was identified and a novel PDX mouse model was established using this case. Subsequent research was conducted to validate the antitumour effects of various FGFR inhibitors on this mutant model [19]. Other groups indicated that aberrant activation of CCDC6 as an independent gene has been implicated in promoting tumour progression in multiple malignancies, including lung, gastric and bladder urothelial carcinoma [20, 21, 22]. Suppression of CCDC6 expression has demonstrated synergistic effects with targeted therapies in ovarian cancer [23]. Subsequently, it has also been identified to have roles as a cellular DNA damage repair element, including facilitating the repair of DNA damage and accomplishing the recognition of DNA damage checkpoints [20, 21, 24]. Besides, CCDC6 was found to be involved in regulating the EMT process in tumours [13]. Nevertheless, the potential role of CCDC6 in the pathogenesis of iCCA remains to be elucidated. Given that CCDC6 has been implicated in DNA damage repair and the regulation of epithelial‐mesenchymal transition in other cancers, we hypothesise that CCDC6 may not only function as an oncogene fusion partner in iCCA but also act as an independent oncogene.

In accordance with the preceding speculative considerations, an initial bioinformatics analysis and tissue microarray technology were employed to examine the expression of CCDC6 as a potential independent factor in hepatobiliary cancer. The results demonstrated that the expression level of CCDC6 was elevated in hepatobiliary ductal carcinoma tissues, and bioinformatics analysis indicated that it may promote carcinoma proliferation and affect prognosis [25]. In this study, we propose to integrate bioinformatics analyses with bidirectional genetic interventions and pharmacological inhibition to further explore the regulatory function of CCDC6 in the malignant progression of iCCA through a series of in vivo and in vitro experiments and multidimensional validation. CCDC6 plays a critical regulatory role in iCCA proliferation, invasion, metastasis and gemcitabine resistance. Its involvement in chemoresistance is driven by dual mechanisms involving EMT and DNA repair pathways, highlighting its significance in both iCCA treatment and prognosis. This hypothesis warrants further mechanistic investigations and validation through clinical translational studies, particularly by recruiting iCCA patients undergoing curative treatment.

## 2 Material and Methods

### 2.1 Public Omics Data Sets and Bioinformatics

Transcriptomic data sets, The Cancer Genome Atlas–cholangiocarcinoma (TCGA‐CHOL), GSE26566 and OEP001105 were analysed as described [26]. Statistical analyses were performed with R v.4.3.0 (R Foundation for Statistical Computing, Vienna, Austria). The linear correlation between CCDC6 expression and KRT19 or MUC1 expression was analysed by the Pearson correlation analysis. *χ* ^2^‐test or Fisher exact test was conducted to evaluate the relationship between CCDC6 expression and clinicopathological TNM stage. Comparison of 2 groups was performed with a 2‐tailed Student t test or Wilcoxon test, and comparison of multiple groups was performed with 1‐way ANOVA. Survival analysis was performed with the Kaplan–Meier method, and the statistical difference was determined by a log‐rank test. The “surv_cutpoint ()” function in the R package survminer was used to determine the optimal cutoff point of CCDC6 mRNA level corresponding to the survival of iCCA patients.

### 2.2 Cell Culture and Transfection

The human intrahepatic cholangiocarcinoma (iCCA) cell lines RBE, HuCCT1 and HCCC‐9810 were acquired from Procell Life Science and Technology Co. Ltd. (Wuhan, China). Cells were maintained in RPMI‐1640 medium (Gibco, Massachusetts, USA) supplemented with 10% fetal bovine serum (FBS) and 1% penicillin–streptomycin solution. Cultures were kept in a humidified incubator at 37°C under a 5% CO₂ atmosphere. The CCDC6 overexpression plasmid, hU6‐MCS‐CBh‐gcGFP‐IRES‐puromycin constructs, and corresponding control vectors were constructed and produced by GeneChem (Shanghai, China) (Table S1). For stable transfection, cells were transfected following standard protocols and then selected with puromycin (2 μg/mL; Sigma‐Aldrich, MO, USA) for 2–4 weeks. For transient transfection, Lipofectamine 3000 (Invitrogen, Carlsbad, CA, USA) was used according to the manufacturer's instructions and previous study [27, 28]. Transfected cells were validated for CCDC6 expression by qPCR and Western blot prior to subsequent experiments.

### 2.3 Western Blot Analysis

Protein was extracted with RIPA buffer (BESTBIO, China) containing protease and phosphatase inhibitors. Protein concentration was assessed by BCA assay kit (Bio‐Rad, USA). Samples were denatured, separated by SDS‐PAGE, and transferred to PVDF membranes (Millipore, USA). After blocking, membranes were incubated in the presence of primary antibodies overnight at 4°C, followed by HRP‐conjugated secondary antibodies. Protein bands were visualised by means of an ECL detection system (e‐BLOT, China). Antibody against CCDC6 (67637‐1‐Ig, 1:5000) was purchased from Proteintech Group (Chicago, IL). Vimentin (#5741, 1:1000), Snail (#3879, 1:1000) and GAPDH (#5174, 1:1000) were purchased from Cell Signalling Technology (Massachusetts, USA). Anti‐mouse and anti‐rabbit secondary horseradish peroxidase (HRP) antibodies were from Bioss (Beijing, China). All strip grey values were calculated using ImageJ (v1.54g) software.

### 2.4 RNA Isolation and Real‐Time qPCR

Total RNA was extracted and purified using the Total RNA Isolation Kit (Vazyme, China) and quantified with NanoDrop (Thermo Fisher Scientific, USA). cDNA synthesis was performed with 1 μg RNA using the PrimeScript RT Kit (Vazyme, China). Real‐time qPCR was performed using a QuantStudio 6 Flex System (Applied Biosystems, USA) with SYBR Green Master Mix (Vazyme, China). Gene‐specific primers (Sangon Biotech, China) were used, and expression levels were normalised to GAPDH using the 2^−ΔΔCt^ method. Triplicate experiments were performed. The primers for the reference gene U6 and target genes, including CCDC6 and GAPDH are listed in Table S2.

### 2.5 Cell Growth Assay

iCCA cells (1 × 10^3^/well) were seeded in 96‐well plates and cultured for 8 days. Cell viability was measured every 24 h using the CCK‐8 assay: 10 μL CCK‐8 reagent was added, incubated at 37°C for 1 h, and absorbance at 450 nm was quantified with a microplate reader (Tecan Trading AG, Switzerland). Triplicate experiments were performed, with data normalised to the initial reading.

### 2.6 EdU Proliferation Assay

EdU staining was completed according to the manufacturer's protocol. 3 × 10^4^ iCCA cells were seeded in 12‐well plates, and one group was treated with 4 μM P5091 (Selleck, Houston, TX) for 24 h based on prior IC50 data. Cells were then incubated with 500 μL EdU working solution (Beyotime, China) at 37°C for 2 h. Using the EdU proliferation kit, proliferating cells (red/green fluorescence) and nuclei of cells (Hoechst 33342, blue fluorescence) were visualised under a microscope (Nikon, Japan). The proliferation rate was assessed as the proportion of EdU‐positive cells to total cells across eight random fields.

### 2.7 In Vitro Wound‐Healing Assay

Cells were cultured in a 12‐well plate and allowed to grow to confluence. They were washed 3 times with PBS, scratched with a 200 μL pipette tip, and microphotographed at 0 h and 24 h under an inverted microscope (magnification, 4×) (OLYMPUS IX73, Tokyo, Japan). Migration Rate (%) = (Initial Scratch Area − Final Scratch Area)/Initial Scratch Area × 100%.

### 2.8 Cell Migration and Invasion Assays

For migration assays, 4 × 10^4^ cells in serum‐free medium were seeded into the upper chamber and incubated at 37°C for 24 h. For invasion assays, Matrigel‐coated chambers (Corning, 356234, USA) were used, and cells were incubated for 48 h. Migrated or invaded cells were fixed with methanol, stained with 0.5% crystal violet, and counted under an OLYMPUS BX63 microscope (Tokyo, Japan) at 20× magnification across eight random fields per filter. Experiments were performed in triplicate.

### 2.9 In Vivo Growth and Metastasis Experiments

In vivo tumour growth was evaluated by subcutaneously injecting 1 × 10^7^ iCCA cells (HuCCT1) in 100 μL PBS into the dorsal region of 5‐week‐old male BALB/c nude mice (*N* = 6). Tumour development was monitored for 6 weeks, after which the animals were euthanised and the tumours were excised for subsequent analysis. Tumour dimensions were measured with callipers and volume was calculated as: volume = length × (width)^2^ × π/6.

For the lung metastasis model, 5‐week‐old male BALB/c nude mice (*N* = 5) received tail vein injections of 1 × 10^5^ iCCA cells (HuCCT1) stably expressing GFP. At 12 weeks post‐injection, mice were euthanised; lung metastases were confirmed by haematoxylin–eosin staining, and metastatic foci were quantified using the IVIS imaging system (LI‐COR Biosciences, Lincoln, NE, USA).

### 2.10 Immunohistochemistry

Tissue sections were deparaffinised with xylene, rehydrated through an alcohol series, and endogenous peroxidase activity was blocked with 3% hydrogen peroxide in methanol for 10 min. Antigen retrieval was performed by incubating slides in EDTA buffer (pH 8.0) and boiling for 5 min. After rinsing in PBS, slides were incubated with primary antibody overnight at 4°C. Subsequently, slides were incubated with HRP‐conjugated secondary antibody (goat anti‐rabbit, 1:100; Invitrogen) for 15 min. Diaminobenzidine was used for detection, followed by counterstaining with haematoxylin.

### 2.11 Construction of the Gemcitabine‐Resistant RBE Cells Model

1 × 10^4^ RBE cells were seeded in 96‐well plates and treated with a gemcitabine (Yeasen, Shanghai, China) concentration gradient (six replicates per group) and PBS (control). After 24 h incubation at 37°C, cell viability was analysed by CCK‐8, and the IC_50_ value (gemcitabine concentration for 50% cell survival) was determined. Surviving cells were selected and cultured with progressively increasing gemcitabine concentrations until stable growth was achieved. The IC_50_ of the resistant model was then re‐evaluated.

### 2.12 Detection of DNA Damage (γ‐H2AX Foci Immunofluorescence)

Cells were seeded on glass coverslips in 24‐well plates and treated as specified. After fixation with 4% paraformaldehyde (Beyotime, China) for 15 min and permeabilisation with 0.1% Triton X‐100 (Sigma‐Aldrich, USA) for 10 min, cells were blocked with 5% BSA (Solarbio, China) for 1 h at room temperature. Cells were incubated overnight at 4°C with anti‐γ‐H2AX primary antibody (Beyotime, China, 1:500), followed by Alexa Fluor 488‐conjugated secondary antibody (Beyotime, China, 1:1000) for 1 h at room temperature. Nuclei were counterstained with DAPI (Beyotime, China) for 5 min. Images were captured using an automated inverted fluorescence microscope (Nikon, Japan). Quantification of γ‐H2AX lesions in at least 8 fields of view per group was performed using ImageJ software (NIH, USA).

### 2.13 Statistical Analysis

Data are summarised and presented as mean ± standard error of the mean (SEM) using GraphPad Prism 9.0 software for analysis. Two‐sample independent *t*‐test is used for two‐group comparisons (e.g., Edu, CCK‐8, wound‐healing, Transwell, Western blot in Figure 2), Tukey post hoc test one‐way ANOVA for multiple groups (e.g., Edu, CCK‐8, wound‐healing, Transwell, Western blot in Figure 3, γ‐H2AX foci immunofluorescence in Figure 4), non‐normal distribution, using Mann–Whitney *U*‐test for two groups or Wilcoxon rank‐sum test for multiple groups (Kruskal–Wallis test for group comparisons) (e.g., Tumour volume, Tumour/body weight, Cell division in Figure 5). Significance was taken as *p* < 0.05.

## 3 Result

### 3.1 CCDC6 Expression Level Associated With TNM Stage in iCCA Patients

**FIGURE 1:**
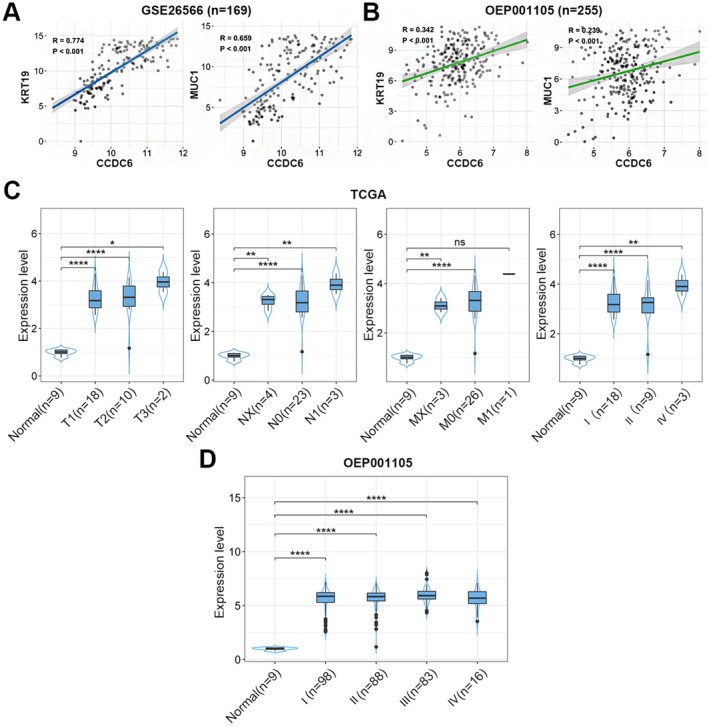
CCDC6 expression level associated with TNM stage in iCCA patients. (A, B) Scatter plots depict the correlation between CCDC6 expression and KRT19 or MUC1 expression in two independent iCCA datasets GSE26566 and OEP001105. Pearson correlation test was used to determine statistical significance. (C, D) Box plots illustrate CCDC6 expression levels in non‐tumour and tumour samples stratified by TNM stage (I–IV) in the TCGA‐CHOL and OEP00105 datasets. Statistical significance was assessed using Bonferroni post hoc correction: ^ns^ *p* ≥ 0.05; *p < 0.05; **p < 0.01; ****p < 0.0001.

Clinical data was collected from iCCA patients in the GSE26566 (*n* = 169), OEP001105 (*n* = 255), and TCGA‐CHOL databases. The results demonstrated that CCDC6 exhibited a positive correlation with the expression of the typical iCCA biomarkers keratin 19 (KRT19) and mucin 1 (MUC1) in the 2 independent iCCA datasets (Figure 1A,B). CCDC6 expression profiles by TNM staging and stratification showed that CCDC6 was increased in patients with advanced iCCA (Figure 1C,D). Although survival analysis did not establish independent prognostic significance (Figure S1), the stage‐dependent expression pattern highlights its clinical relevance in iCCA.

### 3.2 CCDC6 Overexpression Promoted the Proliferation, Invasion and Metastasis of iCCA Cells In Vitro

**FIGURE 2:**
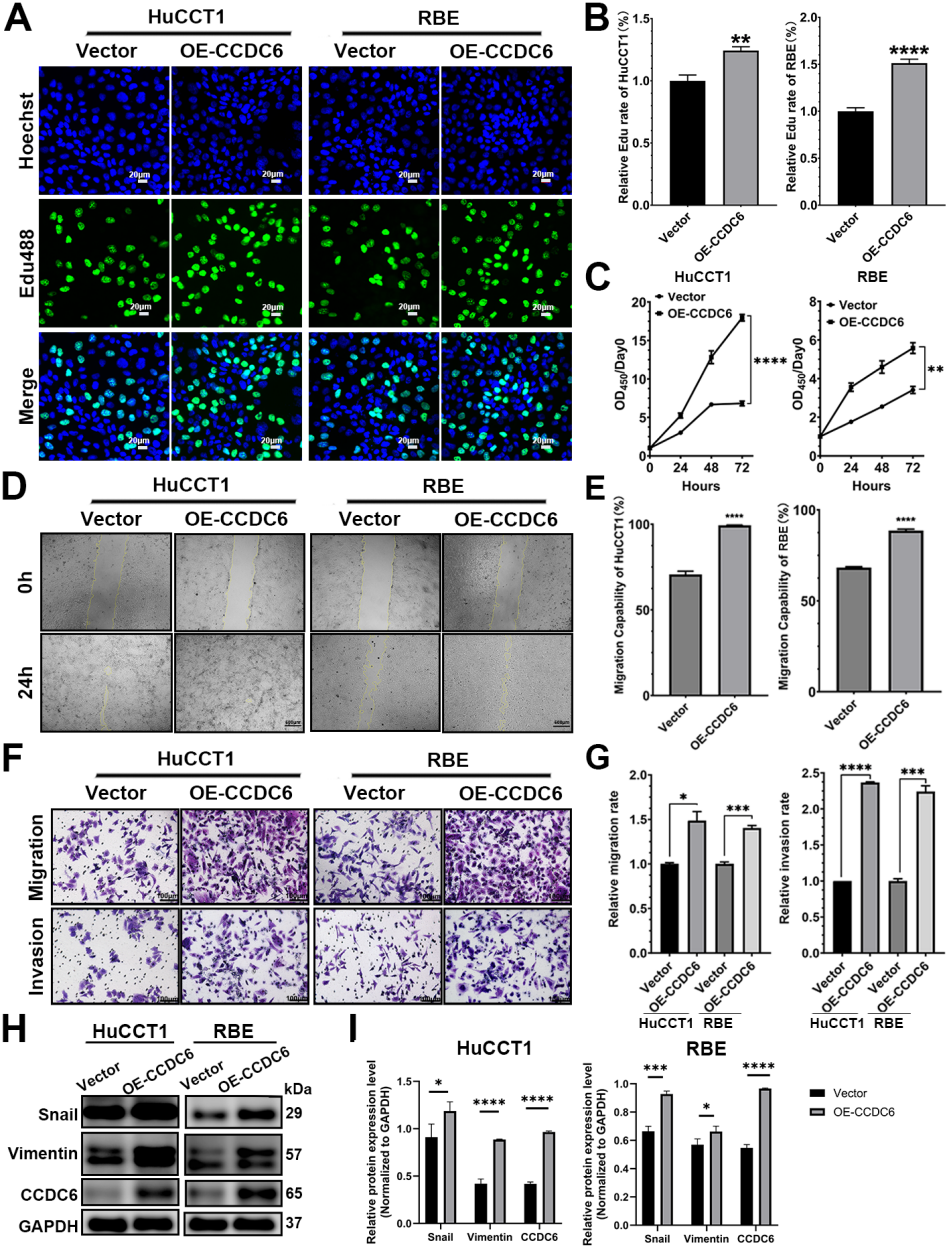
CCDC6 overexpression promoted the proliferation, invasion and metastasis of iCCA cells in vitro. (A) The role of CCDC6 overexpression in iCCA cell proliferation was assessed using EdU proliferation fluorescence staining. Scale bars: 20 μm. (B) Statistical analysis of EdU proliferation fluorescence staining was performed based on fluorescence rates. *N* = 8. (C) The proliferative effect of CCDC6 overexpression was further evaluated by CCK‐8 assay, and growth curves were generated. *N* = 8. (D) Wound healing assays were conducted to compare the migratory capacities of the groups. Scale bars: 500 μm. (E) Statistical analyses of wound healing assays were performed. *N* = 8. (F) Transwell assays (with or without Matrigel coating) were conducted to compare the migratory and invasive capacities of the groups. Scale bars: 100 μm. (G) Statistical analyses of transwell assays were performed. *N* = 8. (H) Western blot analysis was performed to examine the correlation between CCDC6 expression and the EMT marker proteins Vimentin and Snail. (I) Statistical analyses of Western blot analysis were performed. *N* = 3. Vector: Cells transfected with empty vector plasmid. OE‐CCDC6: Cells transfected with a plasmid overexpressing human CCDC6. All data are shown as means ± SEM. *p < 0.05; **p < 0.01; ***p < 0.001; ****p < 0.0001.

To further investigate the regulatory role of CCDC6 in iCCA progression, we established an iCCA cell line overexpressing CCDC6 (OE‐iCCA) via transient transfection with a CCDC6‐overexpressing plasmid. Results from the EdU proliferation fluorescence staining assay demonstrated a significant increase in EdU‐positive fluorescence intensity in CCDC6‐overexpressing (OE) RBE and HuCCT1 cells compared to the Vector control group (Figure 2A,B). Additionally, the proliferation rate (OD_450_) via CCK‐8 assays in the OE group was significantly higher than that in the Vector control group (Figure 2C). In the wound healing assay, OE‐CCDC6 cells exhibited significantly enhanced scratch closure ability, suggesting increased migratory potential compared to the control group (Figure 2D,E). Moreover, Transwell invasion assays revealed a substantial increase in the number of membrane‐penetrating cells in the OE‐CCDC6 group relative to the control (Figure 2F,G).

Furthermore, Western blot analysis demonstrated a significant upregulation of Vimentin and Snail protein expression in RBE and HuCCT1 cells following CCDC6 overexpression (Figure 2H,I). These findings confirmed that CCDC6 overexpression significantly enhances tumour proliferation, invasion and metastasis in vitro by promoting the epithelial‐mesenchymal transition (EMT) process in iCCA cells.

### 3.3 CCDC6 Knockdown Suppressed the Proliferation, Invasion and Metastasis of iCCA Cells In Vitro

To investigate the role of CCDC6 in tumour development, we screened two iCCA cell lines, RBE and HuCCT1, which exhibited the most significant differences in CCDC6 expression among three iCCA cell lines, as determined by qPCR (Figure S2A). A stable CCDC6 knockdown (KD) iCCA cell line was then established using lentivirus‐mediated shRNA, and the knockdown efficiency was validated via qPCR (Figure S2B). Subsequently, we found CCDC6 biological inhibitors used by other researchers through literature review—P5091 [23]. Cell viability was assessed following treatment with P5091 at varying concentrations using CCK‐8 assays. The optimal inhibitory concentration of P5091 was determined to be 4 μM (Figure S2C). Furthermore, the inhibitory effects of P5091 on CCDC6 expression were confirmed at both the mRNA and protein levels through qPCR and Western blot analysis (Figure S2D,E).

**FIGURE 3:**
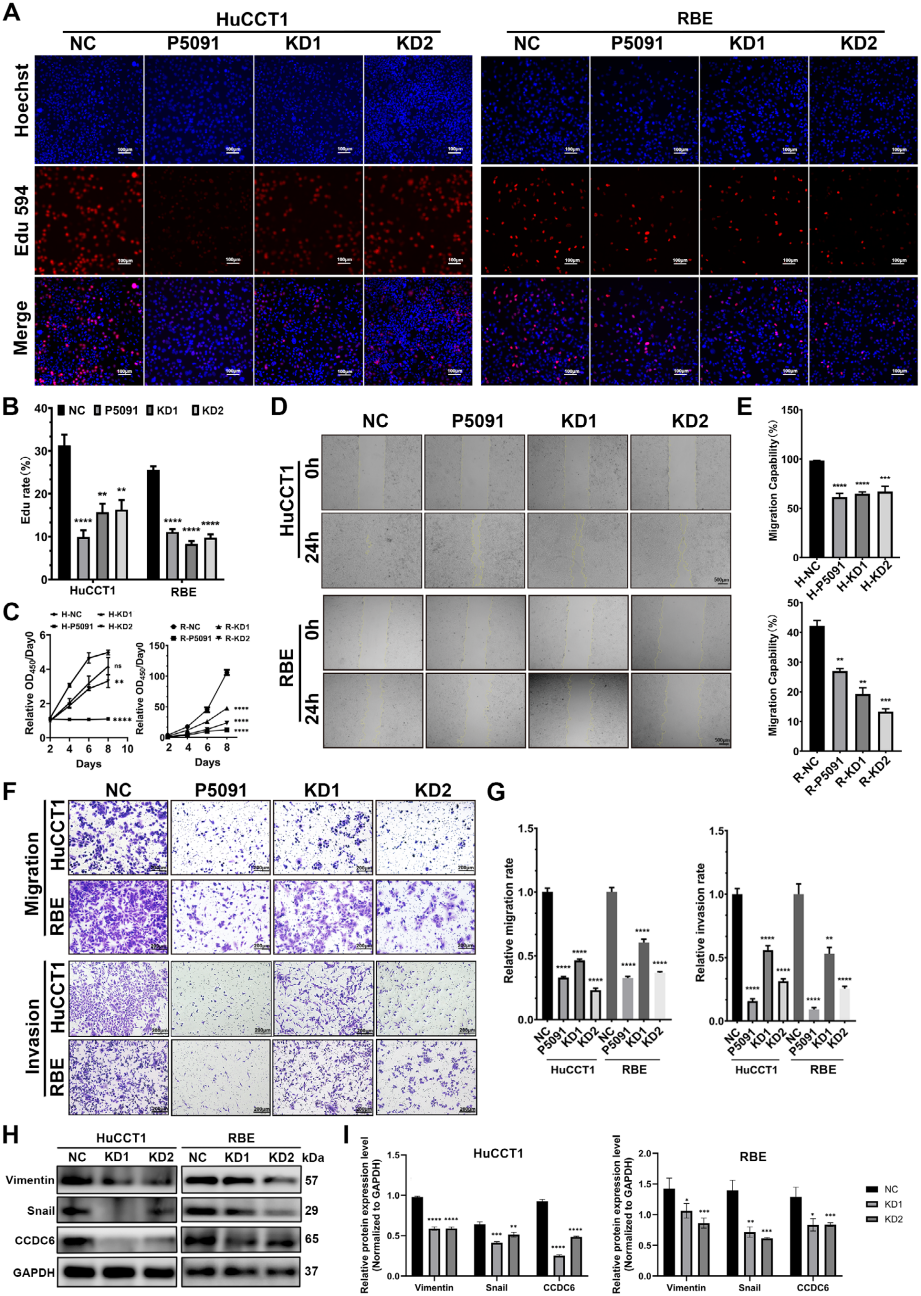
CCDC6 knockdown suppressed the proliferation, invasion and metastasis of iCCA cells in vitro. (A) The effects of CCDC6 knockdown (KD) and P5091 inhibition on the proliferative capacity of RBE and HuCCT1 cells were evaluated using EdU proliferation fluorescence staining. Scale bars: 100 μm. (B) Fluorescence rates of EdU proliferation fluorescence staining were quantified. *N* = 8. (C) Cell proliferation was further assessed using CCK‐8 assays to generate growth curves for the three experimental groups. *N* = 8. (D) Wound healing assays were conducted to compare the migratory capacities of the groups. Scale bars: 500 μm. (E) Statistical analyses of wound healing assays were performed. *N* = 8. (F) Transwell assays (with or without Matrigel coating) were conducted to compare the migratory and invasive capacities of the groups. Scale bars: 200 μm. (G) Statistical analyses of transwell assays were performed. *N* = 8. (H) Western blot analysis was performed to examine the correlation between CCDC6 expression and the EMT marker proteins Vimentin and Snail. (I) Statistical analyses of Western blot analysis were performed. *N* = 3. NC: Cells with non‐targeting control shRNA. KD1: Cells with mild CCDC6 knockdown. KD2: Cells with severe CCDC6 knockdown. P5091: NC cells treated with the CCDC6 inhibitor P5091 (4 μM). All data are shown as means ± SEM. *p < 0.05; **p < 0.01; ***p < 0.001; ****p < 0.0001.

The results of EdU proliferation fluorescence staining demonstrated that EdU‐positive fluorescence in RBE and HuCCT1 cells was significantly weaker in both the CCDC6 knockdown (KD) and P5091‐treated groups compared to the negative control (NC) group (Figure 3A,B). Similarly, CCK‐8 proliferation assays showed that cell proliferation rates, as indicated by OD_450_ measurements, were significantly lower in KD and P5091‐treated RBE and HuCCT1 cells than in the NC group (Figure 3C).

In the wound healing assay, cells in the KD‐CCDC6 groups exhibited significantly impaired scratch closure ability, indicating reduced migratory potential compared to the NC group (Figure 3D,E). Additionally, Transwell migration and invasion assays revealed a substantial reduction in the number of membrane‐penetrating cells in both KD‐CCDC6 groups relative to the control group (Figure 3F,G).

Furthermore, Western blot analysis showed a significant downregulation of Vimentin and Snail protein expression in RBE and HuCCT1 cells following CCDC6 knockdown (Figure 3H,I). These findings revealed that CCDC6 silencing significantly inhibits the EMT process in iCCA while reducing tumour proliferation, invasion and metastatic potential in vitro. Furthermore, this regulatory effect may be dose‐dependent, indicating a potential threshold for CCDC6‐mediated tumour progression.

### 3.4 CCDC6 Overexpression Correlated With iCCA Resistance to Gemcitabine

**FIGURE 4:**
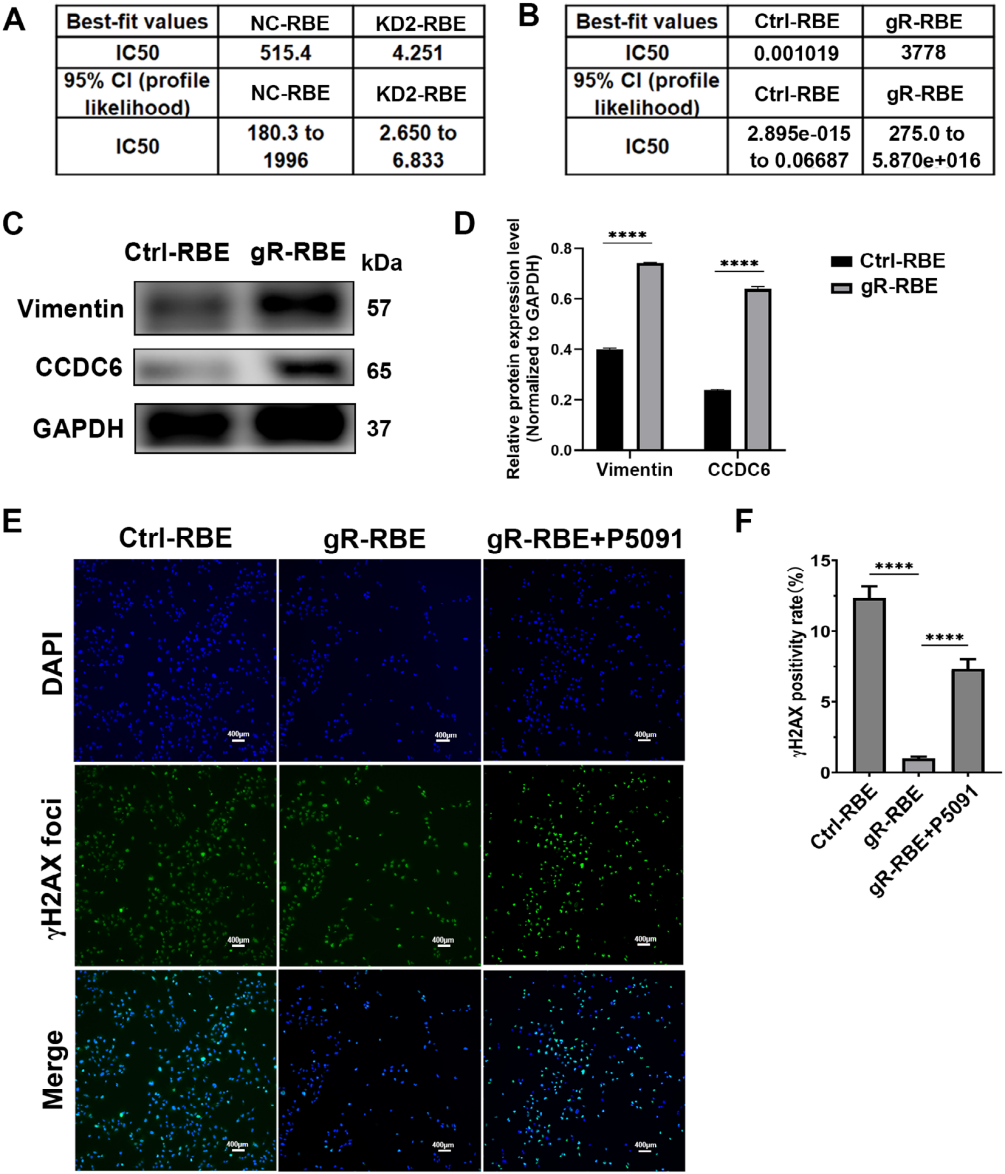
CCDC6 overexpression correlated with iCCA resistance to gemcitabine. (A) CCK‐8 assay was performed to compare gemcitabine resistance between CCDC6‐knockdown RBE cells (KD2‐RBE) and control RBE cells (NC‐RBE). (B) The efficiency of the gemcitabine‐resistant RBE model was validated using CCK‐8 assay. (C) Western blot analysis was conducted to assess the expression of CCDC6 and the EMT marker Vimentin in gR‐RBE cells and Ctrl‐RBE cells. (D) Statistical analyses of Western blot analysis were performed. *N* = 3. (E) DNA damage levels were evaluated by γ‐H2AX foci fluorescence staining in control RBE cells, gR‐RBE cells and P5091‐treated resistant RBE cells. Scale bars: 400 μm. (F) Statistical analysis of γ‐H2AX foci fluorescence staining was performed. *N* = 8. NC‐RBE: RBE cells stably transduced with lentivirus encoding non‐targeting control shRNA. KD2‐RBE: RBE cells stably transduced with lentivirus encoding CCDC6‐specific shRNA to achieve severe knockdown. Ctrl‐RBE: Parental RBE cell line. gR‐RBE: Gemcitabine‐resistant RBE subline generated through prolonged adaptive induction with gradient concentrations of gemcitabine. gR‐RBE + P5091: gR‐RBE cells pretreated with the CCDC6 inhibitor P5091 (4 μM) for 24 h. Values are means ± SEM. ****p < 0.0001.

To investigate the role of CCDC6 in gemcitabine resistance, we first compared gemcitabine sensitivity between NC‐RBE and KD2‐RBE cell lines using CCK‐8 assays. The results demonstrated that CCDC6 knockdown significantly reduced the IC_50_ of gemcitabine, indicating increased sensitivity to the drug (Figure 4A). Then, we established a gemcitabine‐resistant RBE cell line through prolonged gradient adaptive dosing, and CCK‐8 assays confirmed the successful development of a validated gemcitabine resistance model (Figure 4B). Western blot analysis further revealed that gemcitabine‐resistant RBE (gR‐RBE) cells exhibited significantly elevated CCDC6 protein expression, along with an upregulation of the EMT marker Vimentin (Figure 4C,D).

Additionally, γH2AX foci fluorescence staining assays demonstrated that DNA damage levels were significantly lower in gR‐RBE cells compared to control cells. However, pretreatment with the CCDC6 inhibitor P5091 (4 μM, 24 h) effectively reduced CCDC6 expression in gR‐RBE, leading to a marked increase in DNA damage (Figure 4E,F). These findings demonstrated that CCDC6 may contribute to gemcitabine resistance in RBE by modulating both the EMT process and DNA damage response mechanisms, highlighting its potential as a therapeutic target for overcoming chemoresistance.

### 3.5 CCDC6 Knockdown Inhibited the Proliferation and Metastasis of iCCA Cells In Vivo

**FIGURE 5:**
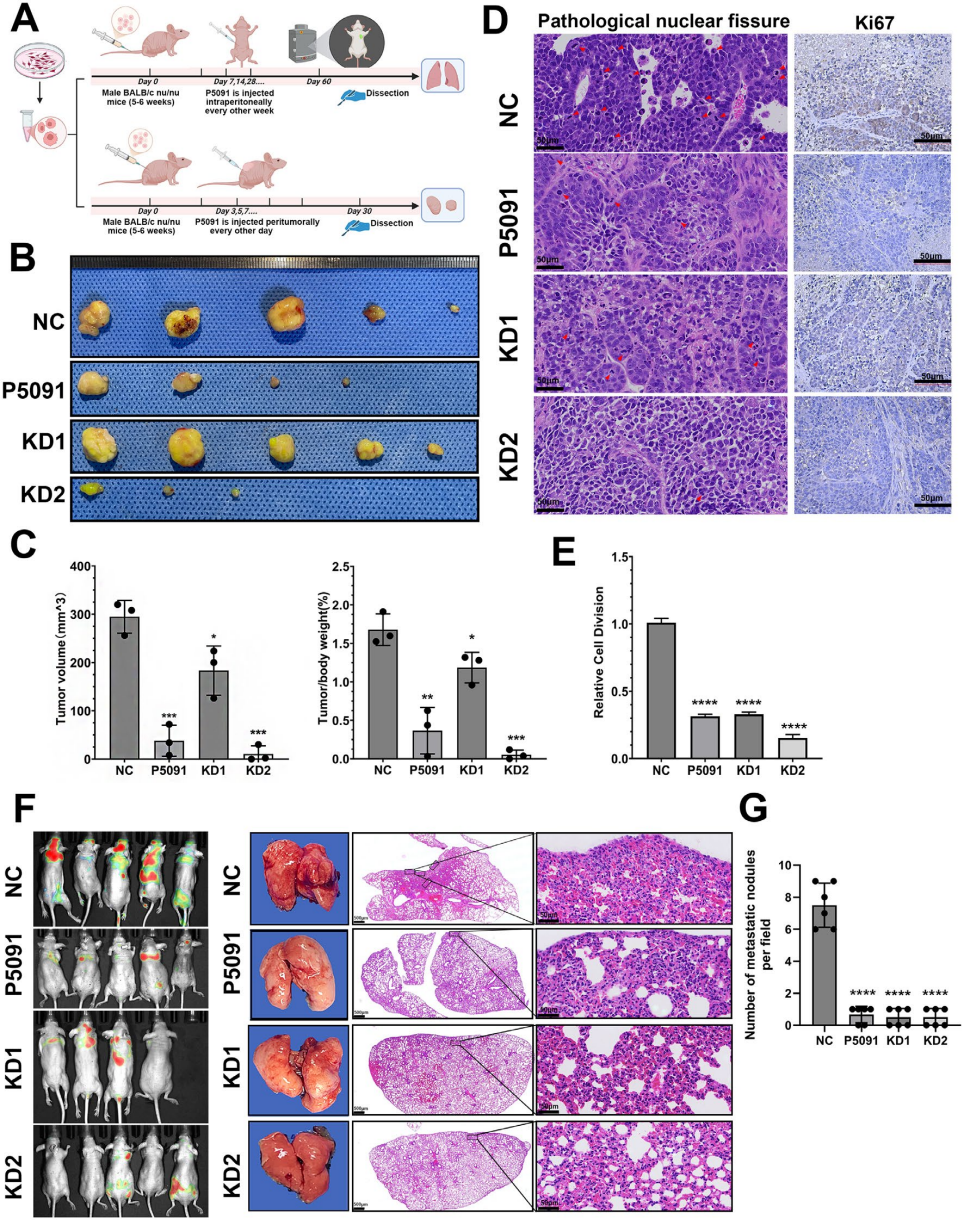
CCDC6 knockdown inhibited the proliferation and metastasis of iCCA cells in vivo. (A) Establishment of subcutaneous xenograft (*N* = 6) and tail vein metastasis models (*N* = 5). (B, C) Tumour volume and weight measurements in the subcutaneous xenograft model and corresponding statistical analysis. *N* = 3. (D) Representative histopathological images of subcutaneous tumours showing pathological mitotic figures and Ki67 immunohistochemical staining. Scale bars: 50 μm. (E) Statistical analysis of pathological mitotic counts across groups. *N* = 10. (F) In vivo imaging of the tail vein metastasis model, representative lung photographs and H&E staining of lung sections to observe metastatic nodules. Scale bars: 500 μm (left), 50 μm (right). (G) The number of lung metastatic nodules in each field of each group. *N* = 6. NC: Mice inoculated with NC control cells. KD1: Mice inoculated with cells exhibiting mild CCDC6 knockdown. KD2: Mice inoculated with cells exhibiting severe CCDC6 knockdown. P5091: Mice inoculated with NC cells and treated with P5091 (20 mg/kg). Values are means ± SEM. *p < 0.05; **p < 0.01; ***p < 0.001; ****p < 0.0001.

To investigate the effects of CCDC6 knockdown on iCCA progression in vivo, we established a subcutaneous tumour formation model (*N* = 6) and a tail vein tumour cell injection model (*N* = 5) in nude mice using NC‐HuCCT1 and KD‐HuCCT1 cell lines (Figure 5A). The results demonstrated that both the number and volume of subcutaneous tumours were significantly lower in the P5091 peritumoral injection group and the KD2 group compared to the NC group, while the KD1 group exhibited a slight reduction in tumour growth (Figures 5B,C and S3). Haematoxylin and eosin (H&E) staining and Ki67 immunohistochemical staining of subcutaneous tumour tissues revealed that the P5091‐treated and KD2 tumours exhibited significantly reduced pathological karyorrhexis and Ki67 positivity rates compared to the NC group, whereas the KD1 group showed only a modest reduction (Figure 5D,E).

Additionally, live imaging and lung tissue histological analysis demonstrated a significant decrease in lung nodule formation in the CCDC6 loss‐of‐function group relative to the NC group (Figure 5F,G). These findings indicated that CCDC6 depletion differentially suppresses the proliferative and metastatic behaviour of iCCA in vivo, with the extent of inhibition correlating with the degree of CCDC6 suppression.

## 4 Discussion

CCDC6 was aberrantly expressed in various malignant tumours, including gastric, lung and ovarian cancers, with its expression closely correlated with tumour progression [18, 20, 21, 24]. Our study elucidated a novel comprehensive regulatory role of CCDC6 in the progression of iCCA. Specifically, CCDC6 overexpression was significantly associated with poor prognosis in iCCA patients and was markedly elevated in cases of advanced disease or lymph node metastasis.

To further delineate the functional implications of CCDC6 in iCCA, we established bidirectional genetic intervention and pharmacological inhibition models. Multidimensional in vivo and in vitro assays demonstrated that modulation of CCDC6 expression significantly influenced tumour cell proliferation, invasion and EMT, thereby driving tumour progression. However, in our study, CCDC6 expression was confirmed in both KD1 and KD2 vectors. We observed that the knockdown efficiency in the KD1 group was lower than that in KD2, which may be attributed to incomplete knockdown, potential off‐target effects, or inherent heterogeneity within iCCA cells. To ensure robustness, two distinct shRNA sequences were used to downregulate CCDC6, and relevant signalling pathways were further investigated. Collectively, these findings position CCDC6 as a promising therapeutic target for iCCA.

Moreover, our study not only expands the current understanding of CCDC6's biological functions but also provides an experimental foundation for the precision treatment of iCCA. Presently, iCCA treatment relies heavily on a regimen of gemcitabine combined with cisplatin, while targeted agents exhibit limited efficacy and are prone to drug resistance. Previous studies have linked both the EMT process and the DDR mechanism to chemoresistance and recurrence in various malignancies, including iCCA [7, 29, 30], yet the potential involvement of CCDC6 in these processes has not been explored.

In our investigation, we initially demonstrated that CCDC6 correlates with gemcitabine chemoresistance in iCCA by comparing loss‐of‐function and overexpression models. Further validation using a gemcitabine‐resistant RBE strain revealed that CCDC6 reduces chemosensitivity by enhancing DDR and by modulating EMT markers, thereby increasing gemcitabine tolerance in iCCA cells. These results not only identified a new target for reversing chemoresistance but also highlighted the translational potential of the small molecule inhibitor P5091. P5091 is a selective inhibitor of USP7, a deubiquitinating enzyme that may maintain its protein levels by antagonising the ubiquitination and degradation of CCDC6. It has been found in studies of colorectal cancer and multiple myeloma to inhibit tumours by regulating pathways such as HDM2‐p53 and Wnt/β‐catenin [31, 32]. However, its role in iCCA has not yet been explored. In this study, we confirmed that reducing CCDC6 expression exogenously using P5091 reverses iCCA tumour resistance to gemcitabine. This provides preliminary theoretical support for combining CCDC6 inhibition with gemcitabine chemotherapy. Despite these promising findings, further investigation is required to fully ascertain the clinical translational value of targeting CCDC6.

In the pathway enrichment analysis of CCDC6 from our previous study [25], we observed that CCDC6 may exert its function through the ErbB pathway in cholangiocarcinoma (CCA). Additionally, in our lab, several molecules associated with CCDC6 levels were preliminarily validated by Western blot analysis (Data not shown). This observation indicates that several classic pathways, such as MAPK and ErbB signalling, may be involved in the malignant progression of CCA. To address current mechanistic gaps, we will analyse multiple bioinformatic databases and establish CCDC6 knockout mice to identify the involved signalling and pathways mediated by CCDC6 in the further study.

Although functional assays have demonstrated the tumour‐promoting role of CCDC6, its mRNA expression did not exhibit significant prognostic value in survival analysis of public cohorts (OPE001105 database, *N* = 244, *p* = 0.19; TCGA database, *N* = 30, *p* = 0.20). Several factors may account for this discrepancy. First, the high heterogeneity of iCCA patients may have obscured its prognostic effect, which could be limited to a specific molecular subtype. Secondly, mRNA levels may not fully reflect the functional activity of CCDC6 protein, as post‐translational modifications and protein stability likely play more critical roles. Additionally, the limited sample size and batch effects inherent in retrospective data may reduce statistical power. Integrating CCDC6 expression data with multi‐centre clinical features using artificial intelligence (AI) and machine learning may contribute to identifying CCDC6‐based individualised strategies for iCCA patients.

## Author Contributions

**Jiaming Chen:** data curation (equal), methodology (equal), writing – original draft (equal). **Qiaoting Wu:** data curation (equal), methodology (equal), writing – original draft (equal). **Bodeng Wu:** data curation (equal), methodology (equal), writing – original draft (equal). **Guanbo Wang:** data curation (supporting), methodology (supporting). **Jiawei Li:** data curation (supporting), methodology (supporting). **Zhenxun Wang:** data curation (supporting), methodology (supporting). **Xinyi Tan:** data curation (supporting), methodology (supporting). **Bo Ma:** data curation (supporting), methodology (supporting). **Xiaoqing Jiang:** writing – review and editing (equal). **Xin Zhang:** supervision (equal), writing – review and editing (equal). **Yu Wang:** conceptualization (lead), funding acquisition (lead), methodology (equal), project administration (lead), writing – review and editing (equal).

## Ethics Statement

Our study was approved by the Animal Ethics Committee of Nanfang Hospital (IACUC‐LAC‐20240325‐003). All procedures performed in studies involving human participants followed the National Research Committee of Nanfang Hospital's guidelines.

## Consent

The authors have nothing to report.

## Conflicts of Interest

The authors declare no conflicts of interest.

## Supporting information


**Figure S1:** The survival analysis of high or low CCDC6 expression levels in two independent databases. (A) The survival analysis of high or low CCDC6 expression levels in OEP001105 databases (*N* = 244). The optimal cutoff point is 50.23456. (B) The survival analysis of high or low CCDC6 expression levels in TCGA databases (*N* = 30). The optimal cutoff point is 3.902463.
**Figure S2:** (A) The differences in CCDC6 expression among three iCCA cell lines were exhibited by qPCR. (B) The knockdown efficiency of the CCDC6 knockdown (KD) iCCA cell lines was validated via qPCR. (C) Cell inhibitory rate was assessed following treatment with P5091, a CCDC6 protein inhibitor, at varying concentrations using CCK‐8 assays. The optimal inhibitory concentration of P5091 was determined to be 4 μM. (D, E) The inhibitory effects of P5091 (4 μM) on CCDC6 expression were confirmed at both the mRNA and protein levels through qPCR and Western blot analysis.
**Figure S3:** Original tumour images in the subcutaneous xenograft model.
**Figure S4:** Uncropped Western blot data.
**Table S1:** The sequence of transfection iCCA Model.
**Table S2:** The sequence of qPCR primers.

## Data Availability

The data analysed in this study are available in the following public repositories: Gene Expression Omnibus (GSE26566), National Omics Data Encyclopedia (OEP001105), and The Cancer Genome Atlas Cholangiocarcinoma (TCGA‐CHOL) cohort.
