# Supplementary material for: Oncogenic Role and Drug Resistance Effect of CCDC6 in iCCA: Potential Strategies for Targeted Intervention
Source: J Cell Mol Med. 2025 Oct 2;29(19):e70855. doi: 10.1111/jcmm.70855 (PMC12489175; doi:10.1111/jcmm.70855)
Supplement: Supplementary file 1 — Figure S1: The survival analysis of high or low CCDC6 expression levels in two independent databases. (A) The survival analysis of high or low CCDC6 expression levels in OEP001105 databases (N = 244). The optimal cutoff point is 50.23456. (B) The survival analysis of high or low CCDC6 expression levels in TCGA databases (N = 30). The optimal cutoff point is 3.902463. Figure S2: (A) The differences in CCDC6 expression among three iCCA cell lines were exhibited by qPCR. (B) The knockdown efficiency of the CCDC6 knockdown (KD) iCCA cell lines was validated via qPCR. (C) Cell inhibitory rate was assessed following treatment with P5091, a CCDC6 protein inhibitor, at varying concentrations using CCK‐8 assays. The optimal inhibitory concentration of P5091 was determined to be 4 μM. (D, E) The inhibitory effects of P5091 (4 μM) on CCDC6 expression were confirmed at both the mRNA and protein levels through qPCR and Western blot analysis. Figure S3: Original tumour images in the subcutaneous xenograft model. Figure S4: Uncropped Western blot data. Table S1: The sequence of transfection iCCA Model. Table S2: The sequence of qPCR primers. [file JCMM-29-e70855-s001.docx]

**Supplementary Figure 1. The survival analysis of high or low CCDC6 expression levels in two independent databases. (A)** The survival analysis of high or low CCDC6 expression levels in OEP001105 databases (N=244). The optimal cutoff point is 50.23456. **(B)** The survival analysis of high or low CCDC6 expression levels in TCGA databases (N=30). The optimal cutoff point is 3.902463.


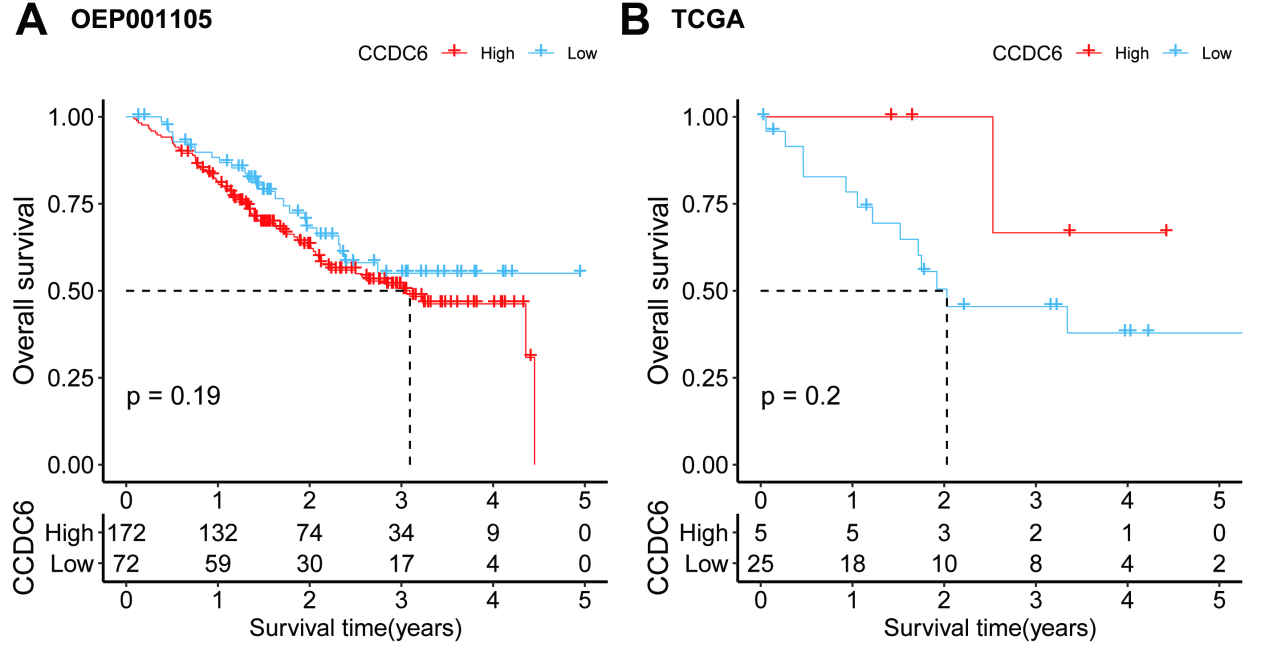


**Supplementary Figure 2. (A)** The differences in CCDC6 expression among three iCCA cell lines were exhibited by qPCR. **(B)** The knockdown efficiency of the CCDC6 knockdown (KD) iCCA cell lines was validated via qPCR. **(C)** Cell inhibitory rate was assessed following treatment with P5091, a CCDC6 protein inhibitor, at varying concentrations using CCK-8 assays. The optimal inhibitory concentration of P5091 was determined to be 4 μM. **(D-E)** The inhibitory effects of P5091 (4 μM) on CCDC6 expression were confirmed at both the mRNA and protein levels through qPCR and Western blot analysis.


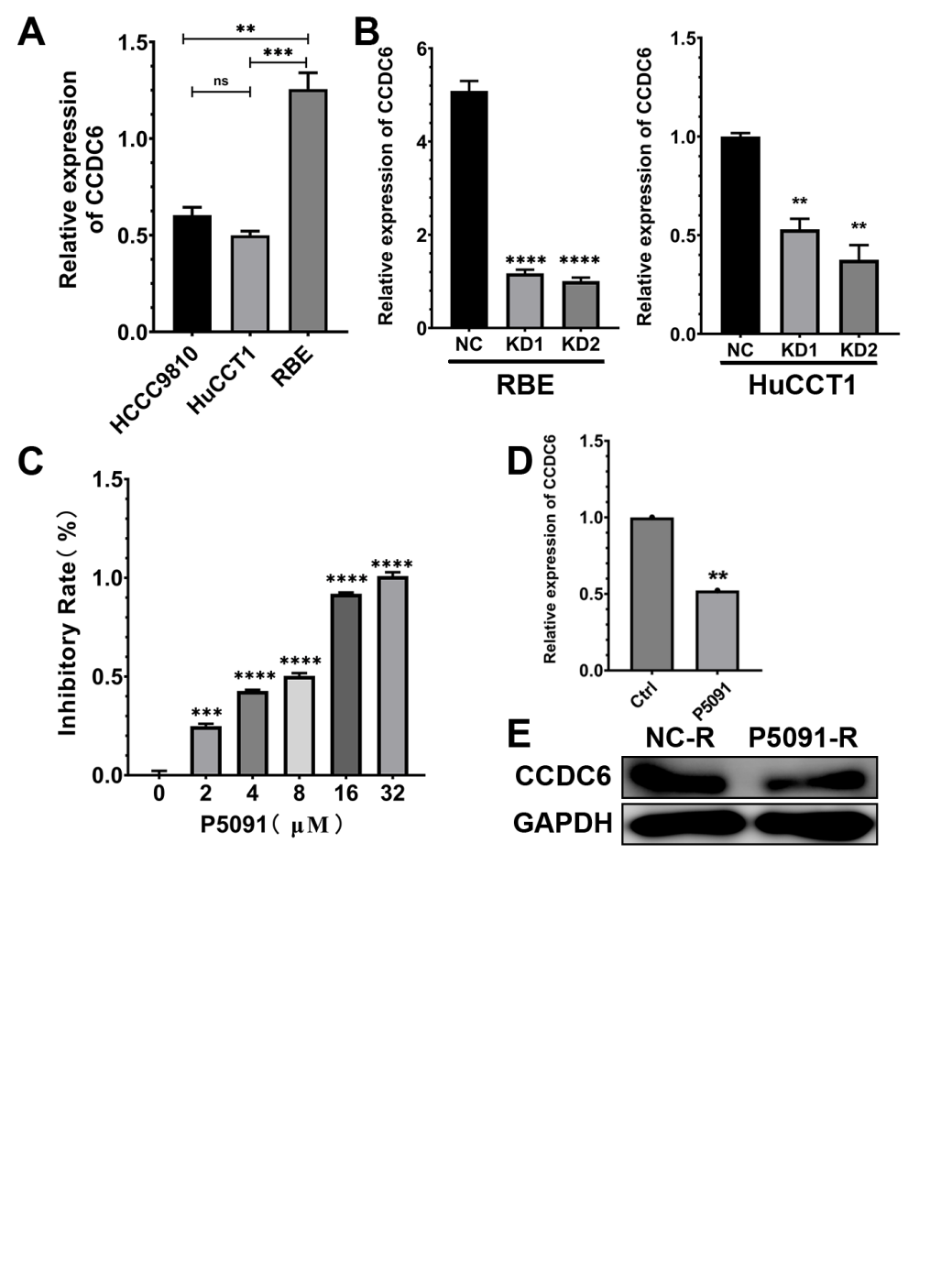


**Supplementary Figure 3. Original tumor images in the subcutaneous xenograft model.**


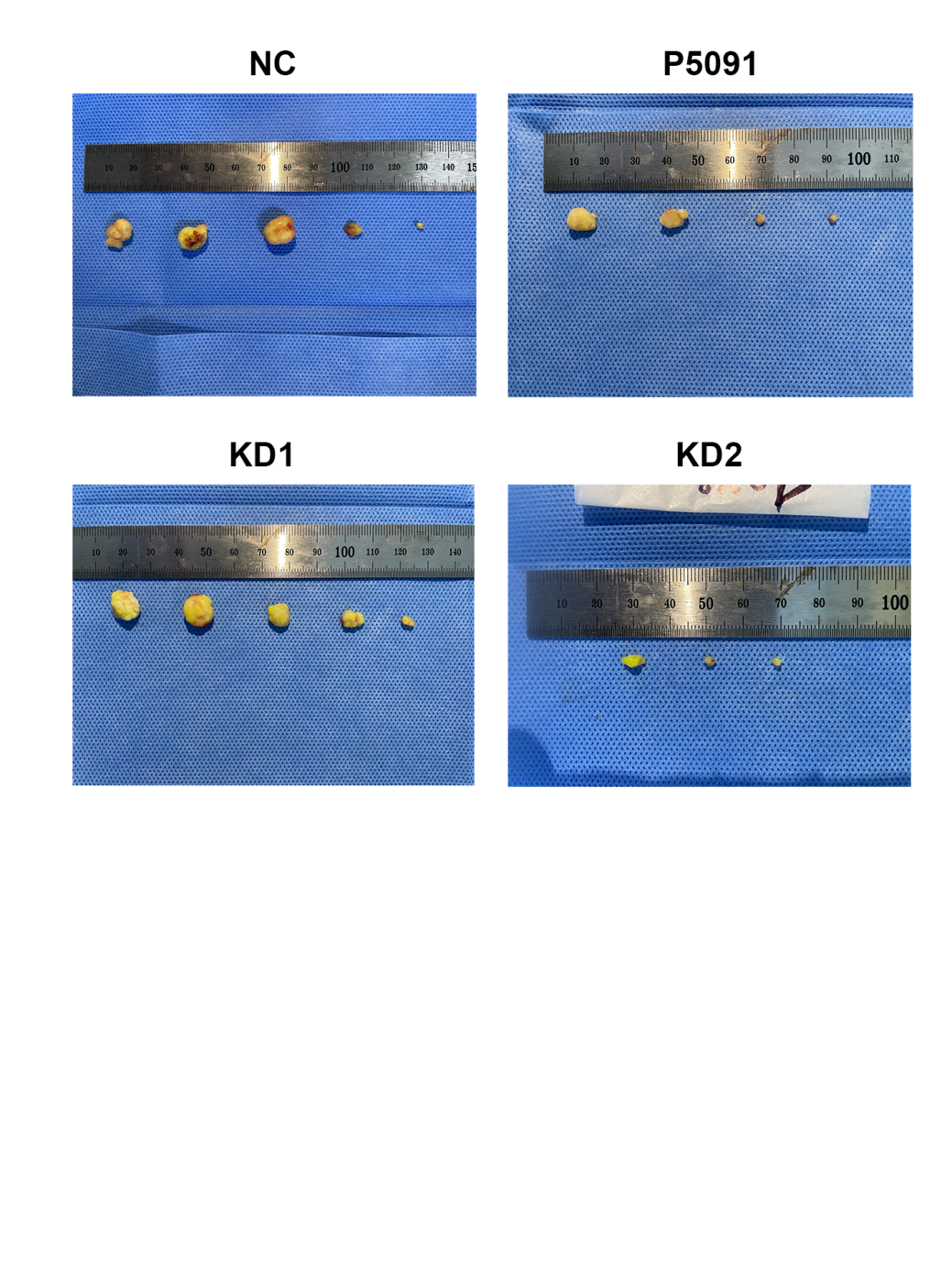


**Supplementary Figure 4. Uncropped Western blot data.**

**
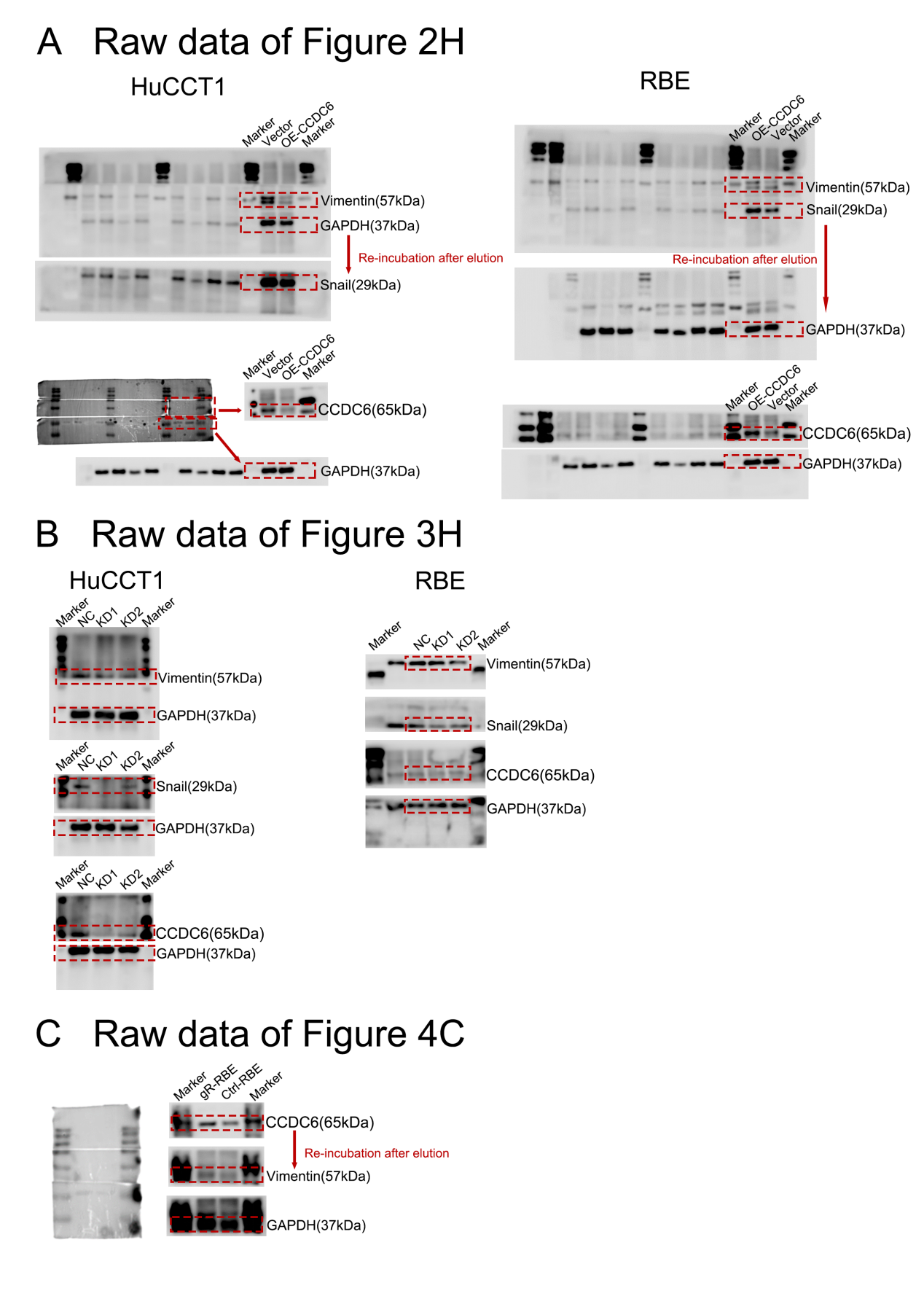
**

**Table S1. The sequence of Transfection iCCA Model**

|  | **Sequence** |
| --- | --- |
| KD1 | GCATGAGAAAGCCGAACTA |
| KD2 | GAACAAGAAGCACTAGTTA |
| OE-CCDC6 | NM_005436（Genbank） |

**Table S2. The sequence of qPCR primers**

|  | **Name** | **Sequence (5’->3’)** | **Position** | **Tm ℃** |
| --- | --- | --- | --- | --- |
| CCDC6 | Forward primer | GTCACACGGTTGGTTTCACG | 1253 | 59.97 |
|  | Reverse primer | CGTGGGCCGTTTGAATTTGT | 1413 | 59.97 |
| GAPDH | Forward primer | AATGGGCAGCCGTTAGGAAA | 58 | 59.96 |
|  | Reverse primer | GCGCCCAATACGACCAAATC | 225 | 59.97 |
